# Low-Rank and Sparse Recovery of Human Gait Data

**DOI:** 10.3390/s20164525

**Published:** 2020-08-13

**Authors:** Kaveh Kamali, Ali Akbar Akbari, Christian Desrosiers, Alireza Akbarzadeh, Martin J.-D. Otis, Johannes C. Ayena

**Affiliations:** 1 Department of Automated Manufacturing Engineering, École de Technologie Supérieure, Montreal, QC H3C1K3, Canada; kaveh.kamali@gmail.com; 2 Department of Mechanical Engineering and the Center of Excellence on Soft Computing and Intelligent Information Processing, Ferdowsi University of Mashhad, Mashhad 9177948974, Iran; akbari@um.ac.ir (A.A.A.); ali_akbarzadeh@um.ac.ir (A.A.); 3 Department of Software and IT Engineering, École de Technologie Supérieure, Montreal, QC H3C1K3, Canada; christian.desrosiers@etsmtl.ca; 4 Department of Applied Sciences, Université du Québec à Chicoutimi (UQAC), Chicoutimi, QC G7H2B1, Canada; martin_otis@uqac.ca

**Keywords:** human gait, recovery, low-rank matrix completion, group-sparsity, missing data

## Abstract

Due to occlusion or detached markers, information can often be lost while capturing human motion with optical tracking systems. Based on three natural properties of human gait movement, this study presents two different approaches to recover corrupted motion data. These properties are used to define a reconstruction model combining low-rank matrix completion of the measured data with a group-sparsity prior on the marker trajectories mapped in the frequency domain. Unlike most existing approaches, the proposed methodology is fully unsupervised and does not need training data or kinematic information of the user. We evaluated our methods on four different gait datasets with various gap lengths and compared their performance with a state-of-the-art approach using principal component analysis (PCA). Our results showed recovering missing data more precisely, with a reduction of at least 2 mm in mean reconstruction error compared to the literature method. When a small number of marker trajectories is available, our findings showed a reduction of more than 14 mm for the mean reconstruction error compared to the literature approach.

## 1. Introduction

Human gait analysis is an important area of study in various fields of application such as the movie and game industry, robotics for trajectory tracking and potential collisions with an operator, medical rehabilitation, prosthetics and sports science, etc. [1]. From different studies summarized in [2,3,4], a large number of body-worn-sensors can be exploited for gait characterization. Among them, optical motion capture systems (MoCap) are standard techniques increasingly used to record walking data [5]. These systems employ two main types of optical technologies such as marker-based MoCap and marker-less-based MoCap. The marker-based technology such as the Vicon system [6] allows tracking of optical markers fixed and thus an accurate gait assessment [7,8]. In this common technique, reflective or LED markers are attached to the body and then tracked by an array of cameras [9]. The 3D marker positions, tracked by such system, can be used in an inverse kinematic model to compute movements of the human skeleton and joint angle trajectories [10]. The marker-less-based technology uses computer vision techniques, including silhouette extraction and skeleton reconstruction in RGB camera for human gait analysis [11,12,13,14,15]. Despite the existence of many sensors available in the literature for gait analysis (their advantages and disadvantages are not belong to the main goal of this study), missing or corrupted data always exist in such systems, and therefore generate an important common problem such as the missing data problem. In the case of MoCap used in this work, the missing data are often a result of occlusion, as markers may be blocked by body parts or other objects during the tracking process [16]. Outliers may also occur when the system confuses one marker with its adjacent markers or reflections of nearby objects. Post-processing of data is usually necessary to recover the missing or corrupted information.

Traditional approaches for this task use linear or cubic spline interpolation to recover gaps in the motion trajectories [17,18]. However, these approaches are only applicable for short duration gaps and can fail to recover longer gaps (more than 200 ms [19]) of missing data. Another common method is the local coordinate system (LCS), which uses redundant kinematic information to reconstruct the motion data [19]. While efficient, this technique requires measured positions of a minimum of three non-collinear markers on each body segment to reconstruct the transformation matrix. In recent years, several methods have also been proposed for the on-line or off-line recovery of missing marker data. Due to their ability to model and predict real-time trajectories, Kalman filters (KF) have been used in various studies for missing data reconstruction [20,21]. However, approaches based on this technique do not fully exploit the inherent properties of human motion. Furthermore, these approaches are limited to the use of some assumptions in which the noise model can be known [22,23,24,25,26]. Thus, KF-based approaches may fail when markers are missing from the beginning or missing partially or entirely for a long period of time [27]. Moreover, the motion data can be corrupted by noise for a long duration. Other methods predict the missing trajectories based on a dataset of similar motions [28]. Despite promising results, these approaches are not adaptive and may fail when recovering trajectories differing from those observed in the training set. A different approach is to estimate missing measurements based on the high correlation of marker trajectories in human motion data [29,30]. One such approach uses principal component analysis (PCA) to fill in the gaps of missing measurements [31]. However, this approach needs long duration tracking data as input (e.g., 20 walking cycles), and does not consider gaps in multiple markers. While an extension was later proposed to recover gaps in multiple markers [29], it still requires a set of uncorrupted trajectories from adjacent markers. Therefore, some new strategies have been proposed to better reconstruct data missing. A number of approaches suggested in the literature, included random sampling techniques, the introduction of influence function techniques, alternating minimization techniques and low-rank matrix recovery [32]. Low-rank matrix completion has recently emerged as a powerful tool in computer vision and image processing to recover missing or corrupted data [33,34,35]. Some researchers [25,36,37,38,39,40,41] used low-rank matrix completion to recover human motion and achieved better recovery results than state-of-the-art methods. Since our proposed algorithm is based on the low-rank matrix technique, we briefly review its formulation in the next paragraph.

The missing entries of a matrix are recovered based on the assumption that the original matrix has a low rank. Let $Y\in R^{m\times n}$ be the incomplete data matrix with subset of observed measurements Ω⊂[m]×[n]. We denote as $P_{\Omega }\left(Y\right)$ the projection of *Y* unto Ω, i.e., the matrix in which observed entries of *Y* are kept and others set to zero. The low-rank matrix completion problem can be formulated as the task of finding matrix $X\in R^{m\times n}$ of lowest rank, such that $x_{ij}=y_{ij}$, ∀(i,j)∈Ω:

(1) $$\underset{X}{arg min}\;\mathrm{rank}\left(X\right),\;\;s.t.\;P_{\Omega }\left(X\right)=P_{\Omega }\left(Y\right).$$

Due to its non-convexity, the rank of a matrix *X* is often approximated using the nuclear or trace norm ${∥X∥}_{*}$, which corresponds to the summation of the singular values of *X*. The nuclear norm has been shown to be the tightest convex approximation of the rank [42]. We note that low-rank matrix completion via nuclear norm minimization can be seen as an extension of PCA, for dealing with missing entries in the data matrix. Missing data from motion requires introducing prior information such as the low-rank. Another regularization prior, commonly used in data reconstruction applications such as compressed sensing, is based on the hypothesis that the data is sparse under some suitable transform [43,44]. We also briefly present this part since our contribution is based on sparse and low-rank matrix completion techniques.

The previous hypothesis could be formulated as the following optimization problem:

(2) $$\underset{X}{arg min}\;{∥\Psi \left(X\right)∥}_{0},\;\;s.t.\;P_{\Omega }\left(X\right)=P_{\Omega }\left(Y\right),$$

where Ψ is the sparsifying transform (e.g., wavelets, Fourier, etc.) and ${∥M∥}_{0}$ is the $l_{0}$-norm, corresponding to the number of non-zeros entries in a matrix *M*. However, due to the non-convexity, research provided the theoretical foundation to transform the non-convex problem caused by $l_{0}$-norm into a convex problem using $l_{1}$-norm. Thus, in practice, the convex $l_{1}$-norm is used to measure sparsity: ${∥M∥}_{1}=\sum_{i,j}\left|m_{ij}\right|$. In some cases, the transformed data Ψ(X) can have a structured pattern of sparsity, where related entries have a greater chance of being simultaneously sparse. This property can be exploited by using a group sparse prior [45], such as the $l_{1,2}$-norm: ${∥M∥}_{1,2}=\sum_{i}{∥m_{i}∥}_{2}$, where $m_{i}$ is the *i*-th row of matrix *M*.

Some studies successfully adopted the low-rank and sparse representation for various applications, such as image repairing [46,47,48,49,50] and action recognition, such as walking, jumping, etc. [51,52,53,54,55,56,57]. Compared to previous works utilizing standard techniques, their reconstruction results showed that a combination of low rank and sparse approach yields significant improvements. Xia et al. [56] designed some experiments including eleven motion such as walking, running, jumping, etc. Their method exploited the property of the low-rank and a sparse representation of the training dataset to predict the missing data, suggesting an efficient recovery of the full-body motion. However, a large number of training sets are needed and it is known that the training process can be time-consuming. A shortcoming is that most previous works in the literature are focused on retrieving the images and human activities recognition. Indeed, most of them, are not directly applicable to the human gait data recovery we cover in this research. When applicable to gait data recovery, these studies often involve the use of a learning method. In this paper, we then propose a new method which leverages recent techniques in these fields. We employ the approach based on low-rank and sparse representation for the recovery of missing measurements in human gait data.

To solve the low-rank and joint-sparse matrix completion problem, augmented Lagrange multipliers (ALM) is the most popular technique applicable to a variety of convex problems. However, our proposed method is based on alternating direction method of multipliers (ADMM) algorithm to optimize the constructed model. The ADMM method is closely related to the ALM but its strategy offers a low computational complexity and a fast convergence rate [58]. Thus, it is used to solve efficiently the local optimization problem. We constructed the nuclear norm regularization minimization model by exploiting the advantage of the low-rank and three different properties. Indeed, the proposed methodology recovers missing data based on three intrinsic properties of tracker trajectories modeling human gait: (1) the high correlation between trajectories of different markers [29,30]; (2) the periodicity and low frequency of these trajectories [59,60,61] and (3) the joint sparse structure of frequencies corresponding to trajectories of different limbs. These properties are used to define a reconstruction model combining low-rank matrix completion of motion data with a group-sparsity prior on the marker trajectories mapped in the Fourier domain. To our best knowledge, it is the first time that a spectrum sparseness and similarity of different gait trajectories are studied. We tested the datasets from [31] and our results reveal that the new proposed method can recover missing marker positions more accurately than the literature approach, in particular when few marker trajectories are available. In addition, using three different types of dataset collected in a Motion Laboratory, we evaluated in this study two different approaches for data recovery by comparing them with an existing method such as the use of PCA. Our findings show that the proposed method outperforms the existing method in terms of the reconstruction accuracy and recovery performance.

## 2. Materials and Methods

We start this section by formulating the problem, including the notation’s definition of the proposed model. We then present our optimization strategy validated by some experiments.

### 2.1. Problem Formulation

**Figure 1:**
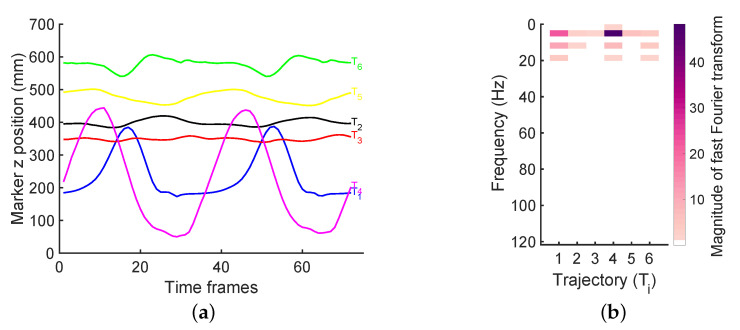
(**a**) An example set of marker trajectories from human gait data. (**b**) The corresponding frequency components.

We suppose the motion capture data consists of the 3D positions of *m* markers at *n* discrete time points. These marker trajectories can be represented as a 3m×n matrix *X*. Moreover, we assume that *X* is only partly observed, with set of observed entries Ω and denote as *Y* the matrix with missing measurements. For the proof-of-principle analysis, we used the available datasets presented in [31], constituted of ten consecutive walking cycles (4300 frames) sampling at 240 Hz. An example of marker trajectories (z coordinates only) is shown in Figure 1a, and contrary to the study in [31], we used a windowed signal for data missing reconstruction analysis. Indeed, the most common method to determine the frequency content of a digital signal is to analyze the frequency spectrum, which can be obtained with the Fourier transform. The digital signal from the human motion changes over time and it is not necessarily stationary. Applying the discrete Fourier transform (DFT) over a long time can not reveal transition in spectral content. Thus, we think that these data over short time could be used in order to represent adequately the frequency content showing more efficient reconstruction’s results than the literature.

By analyzing the trajectories of the markers (Figure 1a), we make the following observations. Firstly, we can see that trajectories of different markers attached to the body are highly correlated. In fact, their *p*-values (p>0.05) indicated that we cannot reject the hypothesis of no correlation between the markers. Moreover, the regression coefficients (r>±0.7) in most cases support our first observation. Consequently, we expect matrix *X* to have a low-rank structure. Secondly, we notice that the trajectories are periodic and mostly composed of low-frequency signals (Figure 1b). Indeed, the frequency of a trajectory is linked to the speed at which the subject moves, and therefore is constrained by biomechanical properties of the body. These biomechanical properties are translated into a sparse representation of trajectories (Figure 1b) with most non-zero components. Finally, we observe that different trajectories share the same frequencies (but possibly different phases and amplitudes), as a result of the synchronized movements of limbs during gait. This third property is conveyed by the joint sparsity of frequencies across different trajectories.

Based on these observations, we propose to use two different formulations for the missing marker recovery problem. The first one proposed, called sparse low-rank (S-LR), imposes two regularization priors on the trajectories, a low-rank nuclear norm and a weighted $l_{1}$-norm sparsity:

(3) $$\left(S-\mathrm{LR}\right)\;\begin{array}{cc} \underset{X}{arg min}\; & {∥X∥}_{*}+\lambda {∥WFX∥}_{1}\\ s.t. & \;P_{\Omega }\left(X\right)=P_{\Omega }\left(Y\right). \end{array}$$

where FX is the fast Fourier transform (FFT) applied on X, with F as a transformation matrix. *W* is a diagonal matrix, such that $w_{ii}$ is a weight corresponding to frequency *i* in the spectrum of *X*. Although the diagonal elements of *W* can be set individually, in this work, we simply give a high penalty to frequency above a predefined threshold, i.e., $w_{ii}=100$ if $i>(\theta_{f}=100)$, else $w_{ii}=1$. These two thresholds are chosen to help promoting sparsity and also to reduce the bias. λ is the hyper-parameter for controlling the trade-off between the low-rank and sparse regularization terms.

In the second proposed formulation, called group-sparse low-rank (GS-LR), we use a $l_{1,2}$-norm to model the joint sparsity of frequencies between the trajectories:

(4) $$\left(\mathrm{GS}-\mathrm{LR}\right)\;\begin{array}{cc} \underset{X}{arg min}\; & {∥X∥}_{*}+\lambda {∥WFX∥}_{1,2}\\ s.t. & \;P_{\Omega }\left(X\right)=P_{\Omega }\left(Y\right) \end{array}$$

Matrix *W* is defined as in the S-LR formulation. Knowing that the rank of a real matrix and the rank of its corresponding Gram matrix are equal [62], we can note $\mathrm{rank}\left(FX\right)=\mathrm{rank}({\left(FX\right)}^{T}FX)$. In addition, applying the transpose matrix properties ($\mathrm{rank}\left({\left(FX\right)}^{T}FX\right)\;=\;\mathrm{rank}\left(X^{T}F^{T}FX\right)$)) and given that $F^{T}F$ is equal to identity matrix ($\mathrm{rank}\left(X^{T}F^{T}FX\right)=\mathrm{rank}\left(X^{T}X\right)$), we then have $\mathrm{rank}\left(FX\right)=\mathrm{rank}\left(X^{T}X\right)=\mathrm{rank}\left(X\right)$ showing the rank preservation property of the Fourier transform. This means that the low-rank prior on *X* also constrains the frequency components of trajectories to be collinear. Thus, constraining the rank of *X* also constrains its rank in Fourier space.

### 2.2. Optimization Strategy

We recover the missing entries of *X* using an optimization method based on the ADMM algorithm [58]. The main idea of this algorithm is to decompose a hard-to-solve optimization problem into easier sub-problems, via constrained auxiliary variables. Using this idea, we introduce variables Q=X and R=FX, and reformulate the problem as:

(5) $$\begin{array}{cc} \underset{X,Q,R}{arg min}\; & {∥Q∥}_{*}\;+\;\lambda {∥WR∥}_{q}\\ s.t. & \;P_{\Omega }\left(X\right)=P_{\Omega }\left(Y\right),\;Q=X,\;R=FX, \end{array}$$

where *q* denotes either the $l_{1}$ norm (S-LR) or the $l_{1,2}$ norm (GS-LR). We then move these added constraints into the cost function using augmented Lagrangian terms with multipliers A,B and corresponding hyper-parameters $\mu_{A},\mu_{B}$:

(6) $$\begin{array}{c} \underset{X,Q,R}{arg min}\;{∥Q∥}_{*}\;+{\;\lambda ∥WR∥}_{q}\;+\;\frac{\mu_{A}}{2}{∥Q-X+A∥}_{F}^{2}\\ \;\;\;+\;\frac{\mu_{B}}{2}{∥R-FX+B∥}_{F}^{2},\;s.t.\;P_{\Omega }\left(X\right)=P_{\Omega }\left(Y\right). \end{array}$$

As mentioned in [58], ADMM-based methods are not overly sensitive to the choice of $\mu_{\cdot }$, these hyper-parameters affecting mostly the convergence of the method. Next, we optimize Equation (6) iteratively with respect to each parameter, until convergence. The following subsections describe how each parameter can be updated.

Updating X: Considering all other parameters fixed, updating *X* corresponds to solving the following least-square problem:

(7) $$\begin{array}{cc} \underset{X}{arg min}\; & \frac{\mu_{A}}{2}{∥X-\left(Q+A\right)∥}_{F}^{2}\;+\;\frac{\mu_{B}}{2}{∥FX-\left(R+B\right)∥}_{F}^{2}\\ s.t. & \;P_{\Omega }\left(X\right)=P_{\Omega }\left(Y\right). \end{array}$$

The optimal solution to this problem is given by:

(8) $$x_{ij}=\left\{\begin{array}{cc} \;\frac{1}{\left(\mu_{A}+\mu_{B}\right)}\left[\mu_{A}\left(Q+A\right)+\mu_{B}F^{T}\left(R+B\right)\right], & \;\;\;\;\;(i,j)\in \Omega \\ \;\;y_{ij}, & \;\;\;\;\;\mathrm{else} \end{array}\right.$$

where $F^{T}\left(R+B\right)$ corresponds to applying the inverse FFT on R+B.

Updating Q: Likewise, *Q* can be updated by solving a nuclear-norm proximal problem given by:

(9) $$\underset{Q}{arg min}{\;∥Q∥}_{*}\;+\;\frac{\mu_{A}}{2}{∥Q-\left(X-A\right)∥}_{F}^{2},$$

the solution of which can be obtained via singular values soft-thresholding [63]. Let $U\Sigma V^{T}=\mathrm{svd}\left(X-A\right)$, we have that:

(10) $$Q\;=\;U\cdot S_{1/\mu_{A}}\left(\Sigma \right)\cdot V^{T},$$

where ${\left[S_{\gamma }\left(M\right)\right]}_{ij}=\mathrm{sign}\left(m_{ij}\right)\cdot \max\left\{|m_{ij}|-\gamma ,0\right\}$ is the soft-thresholding operator.

Updating R: Let C=FX−B, where FX is obtained by applying FFT on *X*. For the S-LR formulation, we update *R* by considering the following $l_{1}$-norm proximal problem:

(11) $$\underset{R}{arg min}{\;\lambda ∥WR∥}_{1}\;+\;\frac{\mu_{B}}{2}{∥R-C∥}_{F}^{2},$$

The solution of this problem is obtained using the soft-thresholding operator:

(12) $$R\;=\;S_{\lambda w_{ii}/\mu_{B}}\left(C\right)$$

Similarly, updating *R* in the GS-LR formulation is given by problem:

(13) $$\underset{R}{arg min}{\;\lambda ∥WR∥}_{1,2}\;+\;\frac{\mu_{B}}{2}{∥R-C∥}_{F}^{2},$$

the solution of which can be computed independently for each row:

(14) $$r_{i}\;=\;\max\left\{∥c_{i}{∥}_{2}-\frac{\lambda w_{ii}}{\mu_{B}},0\right\}\cdot \frac{c_{i}}{∥c_{i}{∥}_{2}}.$$

Updating the Lagrangian multipliers: Finally, we update the Lagrangian multipliers as in the standard ADMM algorithm:

(15) A=A+(Q−X)andB=B+(R−FX).

### 2.3. Experiments and Analysis

We experimentally evaluated the performance of our proposed methods on four different datasets. In this section we first present the data acquisition followed by a description of the data analysis.

#### 2.3.1. Data Acquisition

**Figure 2:**
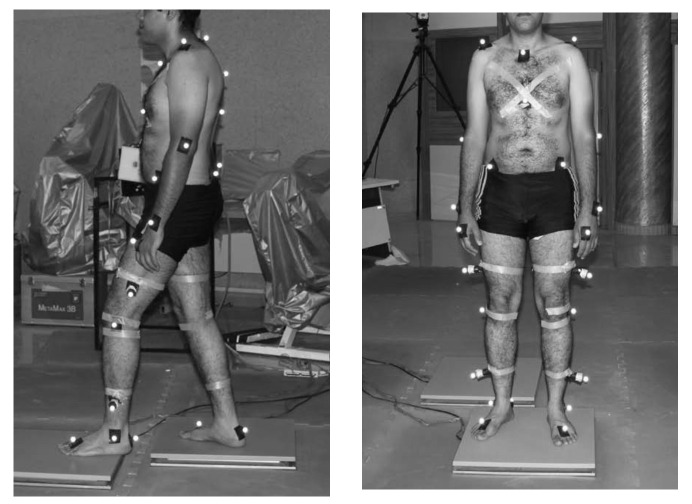
Body-mounted motion capture including 37 markers.

The first three datasets have been recorded on a single healthy adult male, with a mass of 70 kg and a height of 1.72 m. The data were collected in the Motion Laboratory of the Sport Science Research Institute of Iran, equipped with a Raptor-E Digital Real-Time Motion Analysis System. The capture setup is shown in Figure 2. The subject was asked to do a complete gait cycle at three different speeds we denoted by SlowWalk, FreeWalk and FastWalk (from slow walking to running). In addition, we used the freely available dataset (WalkL) of a healthy male adult walking on a treadmill [31]. This dataset (10 consecutive walking cycles) was captured at a frame rate of 240 Hz and contained the x, y, z positions of 37 markers. It was used in [31] as a proof-of-principle for the recovery of gait data.

#### 2.3.2. Data Analysis

We simulated missing capture data by creating synthetic gaps, randomly distributed across markers and time points. For both datasets, 5–20 gaps with a length of 10–50 percent of a complete gait cycle were generated. This procedure was repeated 20 times for each dataset. The accuracy of the proposed methods based on $l_{1}$-norm and $l_{1,2}$-norm sparsity, i.e., S-LR and GS-LR, was evaluated using the average reconstruction error. The reconstruction error is computed as follows as:

(16) $$Error\;=\;|d_{rec}-d_{full}|,$$

where $d_{rec}$ and $d_{full}$ correspond respectively to the Euclidean distance of a marker’s recovered and the true position.

Two different settings are tested in order to evaluate the robustness of the proposed methods with respect to the number of available trajectories. In the first setting, we used the trajectories of all markers for each dataset. In the second setting, we only used one marker trajectory for each human limb, giving a total of 11 different marker trajectories. The two settings are evaluated by applying S-LR and GS-LR approaches to data recovery. These approaches are compared with an existing method such as the use of PCA. Indeed, PCA is the most widely used technique in science and engineering [64]. It is an unsupervised method successfully applied in several studies for identifying characteristics associated with pathological gait, i.e., examining the relation between pathological movement and variability [29,65,66,67,68]. In [31], PCA was used to quantify the interrelation between the movement of different markers. Moreover, tested on long datasets (10 gait cycles), this approach showed better results. For gaps in multiple markers, Gløersen et al. [30] obtained a high reconstruction accuracy using R1 and R2 algorithms based on PCA. The median of their reconstruction results was 2.1 mm and 1.7 mm for the R1 and R2 algorithms, respectively. The spline reconstruction provided a median reconstruction accuracy of 116 mm and therefore was not adequate for recovering highly corrupted datasets. In addition, as showed in [27], the cubic spline performs well over short durations and shows good results. However, for longer gaps, the mean reconstruction error was more than 24 mm. Since we are interested in an unsupervised method and an unbiased data-driven method, PCA, denoted as R2 algorithm in this study, has been chosen as baseline for comparison.

For statistical analysis, Wilcoxon rank sum test is applied to investigate whether there are significance differences between the methods in different conditions.

## 3. Results

**Figure 3:**
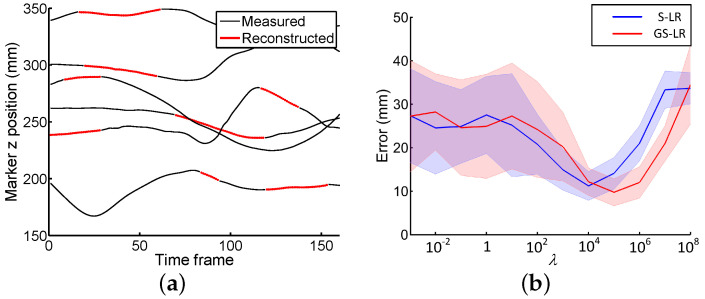
(**a**) A subset incomplete marker trajectories from the WalkL dataset, with recovered measurements shown in red. (**b**) Mean reconstruction error (mm) for different values of regularization parameter λ. Thick lines represent the intra-subject mean profiles and thin lines ±1 standard-deviation margins.

**Figure 4:**
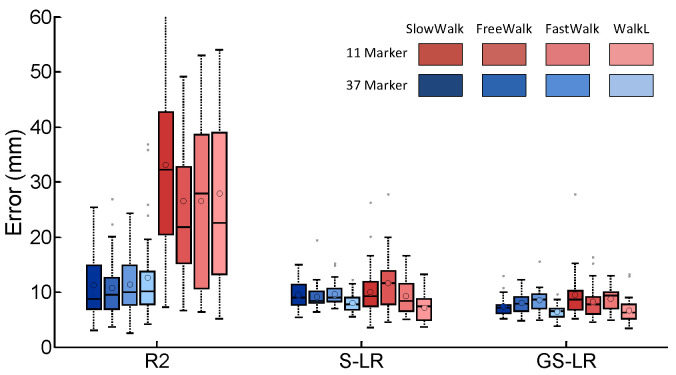
Box plot of reconstruction errors obtained by principal component analysis (denoted as R2), sparse low-rank (S-LR) and group-sparse low-rank (GS-LR) for the 11 marker and 37 marker settings of the SlowWalk, FreeWalk, FastWalk and WalkL datasets.

Figure 3a shows a subset of corrupted trajectories (*z* coordinate only) from the WalkL dataset where measured parts are shown with black lines. The reconstructed parts are based on the GS-LR method. In order to evaluate the impact of the sparse regularization, we reconstructed the WalkL dataset for various values of λ (Figure 3b). Figure 4 illustrates the reconstruction error distributions for the first (use of 37 markers) and second (use of 11 markers) test settings applying three methods. We compared the performance of each method (Table 1) using the pairwise Wilcoxon signed rank test, with a statistical significant set at the 95% confidence level (p<0.05). In Table 1, results indicate that, when all marker trajectories are available, our S-LR and GS-LR methods lead to better accuracy than R2 (p<0.05), with mean reconstruction errors of 10.02 mm and 8.27 mm for S-LR and GS-LR, compared to 12.5 mm for R2. With a total of 11 trajectories, it was 13.8 mm and 12.7 mm for S-LR and GS-LR, respectively, compared to 28.5 mm for R2 (Figure 4).

## 4. Discussion

Most data recovery approaches in the literature are investigated for image repairing [46,47,48,49,50] and action recognition such as jumping [53,54,57]. In this study, we focused on the efficient reconstruction of the missing gait data for various fields of application such as clinical applications (medical rehabilitation, prosthetics, etc.) and sports science, cinema for character animation, robotics for trajectory tracking and potential collisions with an operator in a hybrid work cell, etc. Although the advanced technologies, the missing data problem can be quite challenging. We addressed here this challenge using two different methods called sparse low-rank (S-LR) and group-sparse low-rank (GS-LR). The findings of this study support the underlying intrinsic properties in human motion and show that our proposed method can be used to better reconstruct missing data in signals (Figure 3a).

### 4.1. Comparison of the Two Proposed Approaches for Human Gait Data Recovery

The main contribution of this paper is in four aspects. First, the best data recovery approach (R2) suggested in [30] has been outperformed by using low-rank and sparse representation. Compared to the R2 algorithm, the mean reconstruction error was reduced from 12.5 mm to 8.27 mm. Second, we developed two different approaches, S-LR and GS-LR, for solving the missing data problem in multiple markers. Third, this study evaluated the performance of S-LR and GS-LR approaches on four datasets: SlowWalk, FreeWalk, FastWalk and WalkL. Using these two approaches with 37 markers, the mean reconstruction errors on the WalkL dataset were 8.04 mm and 6.43 mm, respectively which were better than the use of R2 algorithm (12.55 mm). Thus, the findings suggested that our proposed algorithms outperform the literature approach. Moreover, on the other three datasets, R2 algorithm also showed mean reconstruction errors that exceeded 10 mm contrary to our proposed algorithms. Indeed, by comparing the two approaches, GS-LR showed better results on the three datasets with a difference of about 2 mm with S-LR (Figure 4).

In our study, we also evaluated gaps using a smaller number of markers. Using the R2 algorithm, reconstruction of data missing resulted in mean errors more than 20 mm for all the four datasets. However, the mean reconstruction errors with the two proposed approaches provided better results, with errors less than 14 mm (Figure 4). As shown in Figure 4, our methods are robust to the reduced number of trajectories. Indeed, the improvement of S-LR and GS-LR over R2 is more important when less trajectories are available (i.e., one marker trajectory per limb, total of 11 trajectories), with mean reconstruction errors of 13.8 mm and 12.7 mm for S-LR and GS-LR, compared to 28.5 mm for R2. Thus, our numerical simulations compared with R2 algorithm showed better results regardless of the number of markers used and the type of datasets, with a reduction in mean reconstruction errors. Although in Figure 4, a visual comparison between S-LR and GS-LR methods suggests similar accuracies for all datasets, the statistical test (Table 1) indicates that GS-LR leads to smaller errors than S-LR when all marker trajectories are used (p<0.05), while the difference is not statistically significant in the case of limited trajectories (p>0.05 for most datasets). The results were better for the WalkL, which revealed the importance of investigating different types of walking in the solving of, as an example, the pathological gait data recovery problem. In fact, it is known that variability in gait parameters, which can be due to the type of walking (slow, fast or normal), is indicated as an important factor in fall prediction [69]. Accordingly, one of the most important results of this study concern the positive impact of our two proposed methods on the type of walking for various fields of application.

Our main contribution is supported by additional numerical simulations, that allows us to provide a range of optimal values for the parameter λ, which weights the sparse regularization ($l_{1}$-norm and $l_{1,2}$-norms) of the Fourier transform of the matrix *X* (Figure 3b). The results indicated that by adjusting this parameter, the reconstruction result can be improved (Figure 3a). As shown in Figure 3b, we can see that the sparse regularization has a clear impact on the performance of both methods and the best accuracy is obtained for λ between $10^{4}$ and $10^{5}$.

### 4.2. Limitations of This Study

The limitation of this study is the generalization of finding to a wider population, due to the small sample size used. Other methods can also be investigated for more comparison, however, the first evaluation reported here shows encouraging results for new avenues that could be explored in future works. As presented in [56], our proposed methods could also be used with training gait datasets and can provide an improve reconstruction accuracy. However, the training process can be time-consuming and require a large training dataset.

## 5. Conclusions

In this paper, we proposed two methods, based on low-rank and sparse matrix completion, for the reconstruction of missing marker measurements in human gait data exploiting both the low rank as well as its sparsity in a transform domain. We evaluated the accuracy of these methods on four gait datasets (SlowWalk, FreeWalk, FastWalk and WalkL), and compared their performance against a recent approach based on PCA. A statistical comparison of the reconstruction errors show that both S-LR and GS-LR methods outperform this approach, especially when only a single marker is available for each limb. Moreover, results show the GS-LR method to have a better accuracy than S-LR for the full set of marker trajectories, indicating the usefulness of group sparsity for this data. As future work, we will extend these methods not only by capturing the data with elderlies, in particular people with gait disorders, but also including other technologies, methodologies and sources of human gait data, such as foot pressure patterns, electromyographic (EMG) data, etc.

## Figures and Tables

**Table 1:** Performance comparison (*p*-values) of tested methods using a pairwise Wilcoxon signed rank test, for the four gait datasets.

Dataset	GS-LR vs. R2		GS-LR vs. S-LR	
	37 Markers	11 Markers	37 Markers	11 Markers
SlowWalk	0.004	<0.001	<0.001	0.504
FreeWalk	0.022	<0.001	0.034	0.001
FastWalk	0.026	<0.001	0.019	0.688
WalkL	<0.001	<0.001	<0.001	0.094
